# Identification of Genes With Enriched Expression in Early Developing Mouse Cone Photoreceptors

**DOI:** 10.1167/iovs.19-26951

**Published:** 2019-07

**Authors:** Diego F. Buenaventura, Adrianne Corseri, Mark M. Emerson

**Affiliations:** 1 Department of Biology, The City College of New York, City University of New York, New York, New York, United States; 2 Biology PhD Program, Graduate Center, City University of New York, New York, New York, United States; 3 Biochemistry PhD Program, Graduate Center, City University of New York, New York, New York, United States

**Keywords:** cone photoreceptors, single cell, transcriptomic, rods

## Abstract

**Purpose:**

The early transcriptional events that occur in newly generated cone photoreceptors are not well described. Knowledge of these events is critical to provide benchmarks for in vitro–derived cone photoreceptors and to understand the process of cone and rod photoreceptor diversification. We sought to identify genes with differential gene expression in embryonic mouse cone photoreceptors.

**Methods:**

The specificity of expression of the LHX4 transcription factor in developing cone photoreceptors was examined using immunofluorescence visualization in both mouse and chicken retinas. A *LHX4* transgenic reporter line with high specificity for developing mouse cone photoreceptors was identified and used to purify early-stage cone photoreceptors for profiling by single-cell RNA sequencing. Comparisons were made to previous datasets targeting photoreceptors.

**Results:**

The LHX4 transcription factor and a transgenic reporter were determined to be highly specific to early developing cone photoreceptors in the mouse. Single-cell transcriptional profiling identified new genes with enriched expression in cone photoreceptors relative to concurrent cell populations. Comparison to previous profiling datasets allowed for further characterization of these genes across developmental time, species, photoreceptor type, and gene regulatory network.

**Conclusions:**

The *LHX4* gene is highly enriched in developing cone photoreceptors as are several new genes identified through transcriptional profiling, some of which are expressed in subclusters of cones. Many of these cone-enriched genes do not show obvious de-repression in profiling of retinas mutant for the rod-specific transcription factor *NRL*, highlighting differences between endogenous cones and those induced in *NRL* mutants.

Cone and rod photoreceptors are the photosensitive cells of the retina that contribute to image formation. Cones mediate color discrimination and high-acuity vision while rods provide photosensitivity in low-light conditions. Given the importance of cones in high-acuity and color vision, deficiency in this cell type as a result of conditions such as retinitis pigmentosa or macular degeneration leads to a debilitating loss of vision.^1^ As such, the development of cell-based therapeutic strategies based on the formation of new cone photoreceptors is a promising strategy.^2^ However, there is presently a gap in our knowledge of the gene regulatory networks that control the genesis of these cells as well as the early steps in their differentiation. Thus, the design of informed strategies to direct cone production and ways to appropriately benchmark the differentiation of de novo generated cells is lacking.

Two main strategies have been used to investigate the early gene regulation programs of cone photoreceptors. One has been to develop reagents to label developing cone cells or the retinal progenitor cells (RPCs) that generate them and use high-throughput methods to identify the genes with enriched expression in these cells compared to other cells present at the time.^3^,^4^ Such methods have also been used at later differentiation timepoints.^5^–^7^ While these methods provide critical information, they also rely on transcriptional reporters that may have expression that is broader than just cone photoreceptors and do not provide cellular resolution of these gene expression patterns. The second strategy to examine cone photoreceptor gene expression has been through the use of the neural retina leucine zipper (*NRL*) mouse model. *NRL* is a critical rod-expressed transcription factor that is necessary to promote rod gene expression and repress cone gene expression in rod cells.^8^ The *NRL* mouse knockout leads to a large increase in cone gene expression as the large number of rods in the mouse undergo de-repression of cone genes.^9^–^11^ These *NRL* knockout rods have been interpreted as either a complete fate switch to cones or a partial conversion to “cods.”^8^,^12^,^13^ As these cells have been used in a number of studies to model cone photoreceptors, the extent to which these cells are transformed to the cone fate is important both for a justification in using them as a model for endogenous cone cells and to understand photoreceptor diversification.

Here, we identified the LIM homeobox protein 4 (*LHX4*) gene as enriched in cones during early chick retinal development, in addition to bipolar cells (BCs). In the mouse, LHX4 was also determined to be a reliable marker for cones during early retinal development but with expanded expression in late embryonic development and eventual expression in BCs. A *LHX4::GFP* transgenic line^14^ recapitulated the endogenous LHX4 expression pattern and was used to generate a single-cell dataset highly enriched in cone photoreceptors, which provided an in-depth look at the molecular profile of these cells in the earliest stages of differentiation in the mouse. A comparison was made between previous datasets that targeted mammalian cones and photoreceptors, including those made in the *NRL* knockout mouse. This led to the identification of unique cone expression signatures not observed in previous datasets, including those of *NRL* knockout rods, supporting previous observations that these cells are not completely transformed into cones.

## Materials and Methods

### Animals

Experiments involving animals were in accordance with animal care protocols of the City College of New York, CUNY (IACUC-approved protocols 965 and 932), and the ARVO Statement for the Use of Animals in Ophthalmic and Vision Research. The *LHX4::GFP* strain is a bacterial artificial chromosome (BAC) insertion and (strain: Tg(Lhx4::EGFP)KN199Gsat/Mmucd, RRID:MMRRC_030699-UCD) was obtained from the Mutant Mouse Resource and Research Center (MMRRC) at University of California Davis (Davis, CA, USA). *LHX4::GFP* mice were kept and used experimentally only as heterozygotes. Charles River (Kingston, NY, USA) provided CD-1 mice and chick eggs already fertilized, which were kept in a 16°C room for up to 10 days. Eggs were incubated in a 38°C humidified incubator for 5 days before use.

### Genotyping

Genotyping was performed as specified in the MMRRC strain description page referenced (*LHX4::GFP* strain: Tg(Lhx4::EGFP)KN199Gsat/Mmucd, RRID:MMRRC_030699-UCD).

### Plasmids and Electroporation

Misexpression plasmids were created by PCR amplifying the coding sequence for each gene with added *Age*I/*Nhe*I or *Eco*RI sites. They were subsequently cloned into a CAG backbone, created after Stagia3^15,16^ had enhanced green fluorescent protein (*EGFP*) removed and the CAG promoter cloned upstream of the original *EGFP* region. For mouse *LHX4* the primers were designed against the annotated mRNAs in NCBI galgal5 RNA libraries with an added Kozak sequence 5′ (ACC). For 5pLHX4GFP, the 5′ primer from genotyping for *LHX4::GFP* mice was combined with a 3′ primer to the coding region of GFP and the fusion gene was amplified from genomic DNA extracted from *LHX4::GFP*+ mice. CAG::nucβGal was developed by the Cepko lab (Harvard Medical School). To deliver the plasmids after retinal dissection, ex vivo electroporation experiments were carried out as detailed by Emerson and Cepko.^16^

### Tissue Dissociation and Flow Cytometry

Dissociation of retinal tissue and fluorescence-activated cell sorting (FACS) were performed as in Buenaventura et al.^3^

### Immunohistochemistry

All tissue processing and immunofluorescence experiments were performed as in Emerson and Cepko^16^ and Buenaventura et al.^3^ Antibodies used are the following [antibody (host, vendor, catalogue #, dilution, RRID)]: anti-Otx2 (goat, Novus Biologicals, Centennial, CO, USA; AF1979, 1:500, AB_2157172), anti-Rxrg (mouse, Santa Cruz Biotechnology, Dallas, TX, USA; sc-365252, 1:50, AB_10850062), anti-Lhx4 (rabbit, Proteintech, Rosemont, IL, USA; 11183-1-AP, 1:250, no RRID), anti-Lhx3/4 (mouse, Developmental Studies Hybridoma Bank [DSHB], Iowa City, IA, USA; 67.4E12, 1:200, AB_2135805), anti-Nr2e3/PNR (mouse, R&D Systems, Minneapolis, MN, USA; PP-H7223–00, 1:250, AB_2155481), anti-β-galactosidase (mouse, DSHB; 40-1a-s, 1:20, AB_528100), rabbit anti-PKC-alpha (rabbit, Sigma-Aldrich, St. Louis, MO, USA; P4334, 1:500, AB_477345), anti-MAFA/L-MAF (rabbit, gift from Celio Pouponnot, 1:500, no RRID), anti-RAXL (rabbit, gift from Hajime Ogino, 1:500, no RRID), anti-Cone Arrestin (rabbit, Millipore Sigma, Burlington, MA, USA; AB15282, 1:2,000, AB_1163387), and anti-GFP (chicken, Abcam, Cambridge, MA, USA; ab13970, 1:2000, AB_300798). RRID = Repository Resource ID from The Antibody Registry (http://antibodyregistry.org, in the public domain).

### EdU Pulse and Detection

Pregnant dams were injected intraperitoneally with 150 μL 10 mM 5-ethynyl-2′-deoxyuridine (EdU) resuspended in 1× phosphate-buffered saline (PBS) and euthanized 2 hours later to obtain embryonic day 14.5 (E14.5) retinas. Postnatal day 0 pups were injected subcutaneously with 15 μL 4 mM EdU resuspended in 1× PBS. Click-iT EdU Alexa Fluor 647 imaging kit (C10340, Invitrogen, Carlsbad, CA, USA) was used to detect the EdU+ cells.

### Micrographs

Micrographs were taken using a Zeiss LSM880 confocal microscope (Zeiss, Oberkochen, Germany) and ZEN Black 2015 2.1 SP2 software. Fiji was used to convert images between formats.^17^ Panels and figures were constructed using Affinity Designer vector editor (Serif [Europe] Ltd., Nottingham, UK). Images were only adjusted for brightness and contrast, uniformly across the micrograph.

### 10× Single-Cell Sample Processing

Live cells were collected after dissociation and flow sorting for GFP signal.^3^ Samples were extracted from two litters of E14.5 LHX4::GFP mice, pooled into one sorting solution during dissociation. For each sample (GFP+ and GFP−), approximately 4000 cells were individually lysed, and their RNA transcribed and sequenced by the Columbia University Single Cell Analysis Core. Filtered count matrixes provided through the Cellranger version 2.1.1 pipeline (10x Genomics, Pleasanton, CA, USA) using the refdata-cellranger-mm10-2.1.0 genome were used for downstream analysis.

### Single-Cell Clustering Analysis

Unsupervised clustering analysis was performed using Seurat.^18^,^19^ Single-cell transcriptomes from both *LHX4::GFP* E14.5 samples were analyzed in concert to produce cluster and t-distributed stochastic neighbor embedding (TSNE) analysis. Only cells with over 200 genes detected and only genes detected in more than three cells were used for initial loading of the cell matrices. Cell cycle was scored and was regressed out as an unwanted source of variation according to Seurat's guidelines.^20^ For the *LHX4::GFP* dataset, cells were filtered for maximum 3500 and minimum 250 number of genes, and <0.25% mitochondrial content following standard guidelines for Quality Control (QC). nUMI, nGene were regressed out in addition to cell cycle. Using the established jackstraw procedure^21^ in Seurat, 30 principal components (PCs) were chosen for clustering and TSNE projections. The FindMarkers function was used with default parameters, only for positive markers, with the populations compared as described in Results. For Clark et al. similar procedures were used with the following changes: for the full dataset, 250<nGenes<3500, 0.075<percent mito, 39 PCs and replicate effects were regressed out; for the photoreceptor subclustering, same as for full but 6 PCs, data were extracted post analysis of the full dataset.

### Data Comparisons

For the comparison of retinal datasets, differential expression output data were obtained from each report.^4^,^7^,^22^ The data were cross-referenced to the genes obtained in the analysis from this paper and are detailed in Supplementary Files S3–S6. Thresholds for statistical significance corrected for multiple and for each dataset were established as <0.05 for adjusted *P* values (p_val_adj) or *q* values. In the case of Kim et al.,^22^ genes are reported with posterior probability of differential expression (PPDE) as probability of differential expression and were filtered to show only those with PPDE > 0.95.

### Data Availability

Transcriptome data will be deposited in NCBI's Gene Expression Omnibus^23^ (GEO) and will be accessible through GEO Series accession number GSE132272.

### Counting of Markers in Retina Sections

One z-stack from the central portion of each retina of four biological replicates was counted and used for mean and standard error of the mean (SEM) calculations for each timepoint. ImageJ or Fiji and the Cell Counter extension were used.^17^,^24^

## Results

### LHX4 Is Present in Early Cone Photoreceptors in the Chicken Retina

**Figure 1:**
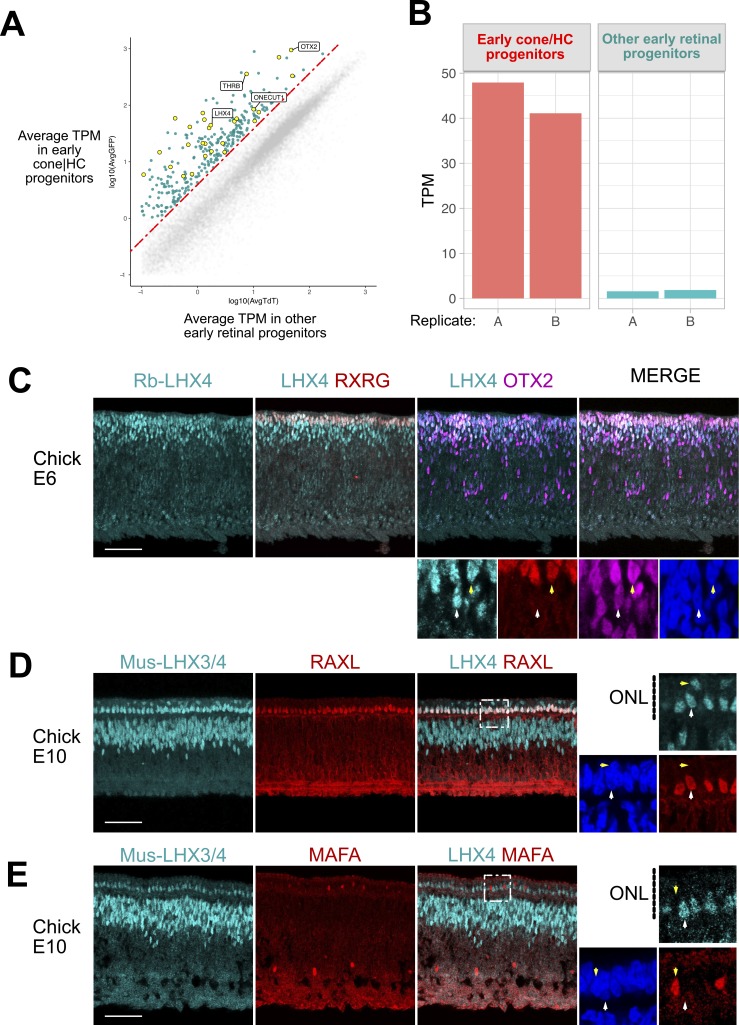
LHX4 is present in early cone photoreceptors during chick retinal development. Large: maximum-intensity projection Z-stacks; small: single planes of the same Z-stacks. (A) Average transcripts per million (TPM) of genes between cone/HC progenitors and other early retinal progenitors. Highlighted in green are genes 1.5-fold enriched in cone/HC progenitors and highlighted in yellow are transcription factors. (B) TPM values for LHX4 in cone/HC progenitors and other early retinal progenitors. (C) Cross section of E6 chick retina imaged for LHX4, RXRG, and OTX2. Higher magnification: a single z-plane with marked LHX4+/OTX2+ (white arrow) or LHX4+/OTX2+/RXRG+ cell (yellow arrow). (D) Cross section of E10 chick retina imaged for LHX4 and RAXL. Higher magnification: a single z-plane with marked LHX4+/RAXL+ (white arrow) or LHX4+/RAXL− cell (yellow arrow). (E) Cross section of E10 chick retina imaged for LHX4 and MAFA. Higher magnification: a single z-plane with marked LHX4+/MAFA− (white arrow) or LHX4−/MAFA+ cell (yellow arrow). Scale bar represents 50 μm.

Recently, we established the transcriptional profile of RPCs, defined by the activity of the ThrbCRM1 cis-regulatory element, that are biased toward the cone and horizontal cell (HC) fate in the early chick retina.^3^ Using this dataset, we screened for potential cone-enriched transcripts that could serve as markers for early cones. After establishing a criterion for >1.5-fold change score between cone/HC RPCs and other concurrent populations (enriched in “other early retinal progenitors”) we selected for transcription factors (TFs) enriched in the cone/HC RPCs (Fig. 1A). We identified the *LHX4* gene as highly enriched, along with known TFs in this population such as *THRB*, *ONECUT1*, and *OTX2*. This transcript has significant fold change (*b* = 3.3) and a low number of reads in the non-ThrbCRM1 active population, suggesting high specificity toward the cone/HC RPC population at this time (Fig. 1B).

A previous report examined the presence of LIM-domain factors in early chick photoreceptor development.^25^ This study suggested that LIM homeobox protein 3 (LHX3) was abundantly present in the apical portion of the retina and localized to photoreceptors once the outer nuclear layer (ONL) is clearly distinguished. As the RNA-Seq data indicated that *LHX4* expression is prominent in ThrbCRM1 reporter-positive cells at early stages while *LHX3* transcript presence is marginal in all targeted cells (Supplementary Fig. S1A), we suspected that this previous study could have detected LHX4 instead of LHX3 at earlier timepoints. To test this, we electroporated a mouse *LHX4* misexpression plasmid (CAG::mLHX4) alongside a CAG::nucβGal construct into the chick E6 retina, cultured it for 2 days, and detected for LHX4 using the LHX3 DSHB antibody. As predicted, we observed robust immunoreactivity of βgal+ electroporated cells with the LHX3 antibody (Supplementary Fig. S1B), suggesting that this antibody (hereafter referred to as LHX3/4 antibody) is also capable of detecting mouse LHX4, and thus likely chicken LHX4 as well.

With the use of this LHX3/4 antibody and a rabbit polyclonal antibody, we examined LHX4 presence during embryonic development of chick at E6 and E10. Expression at E6 is restricted to the scleral portion of the retina, where photoreceptors are located. As the *LHX4* transcript was highly enriched in cells that activate the ThrbCRM1 element, which requires orthodenticle homeobox 2 (OTX2) for activity, we expected LHX4 to be present predominantly in the OTX2+ population at E6. Indeed, LHX4 is detected in a large percentage (but not all) of OTX2-positive cells (Fig. 1C).

At E6, cones are the major class of photoreceptors that are produced, as the earliest known rod photoreceptor marker in the chicken retina, L-Maf (MAFA), is not detected until E9.^26^ As the LHX4 pattern at this stage is localized in the apical portion of the retina, we examined LHX4+ cells for coexpression with the cone marker retinoic acid receptor RXR-gamma (RXRG). Many of the most apically located LHX4 cells were indeed positive for RXRG (Fig. 1C).

It is unclear to what extent and in which cells LHX3, LHX4, or both proteins are present at later timepoints when the transcriptional status of *LHX3* may change. In one report,^25^ antibody staining using DSHB anti-LHX3/4 showed strong nuclear ONL and inner nuclear layer (INL) signal at E10, which suggested photoreceptor and bipolar signals. We also detected signal in the ONL and INL with the LHX3/4 antibody. However, the rabbit anti-LHX4 antibody detected a similar pattern with strikingly strong signal in the ONL and weaker in the INL (Supplementary Fig. S1C). As clear evidence of *LHX3* RNA expression in the ONL was not observed in the previous study, this suggests that *LHX4* and not *LHX3* may continue to be expressed in E10 chicken photoreceptors.

To examine the photoreceptor subtype expression more closely, we compared to retina and anterior neural fold homeobox 2 (RAXL) expression, a marker for cone photoreceptors,^26^ and observed that all RAXL-positive cells were also positive for LHX3/4 (Fig. 1D). A smaller number of cells in the upper part of the ONL were LHX4-positive and not RAXL-positive. As the ONL contains both cones and rods, we used an antibody to MAFA,^26^,^27^ the earliest rod marker, to determine if these cells were rod photoreceptors. No overlap between MAFA and LHX3/4 was detected at E10 in the ONL (Fig. 1E). These data suggest that LHX4 is expressed predominantly in developing cone photoreceptors in the chicken retina.

### LHX4 Is Present in Early Cone Photoreceptors in the Mouse Retina

**Figure 2:**
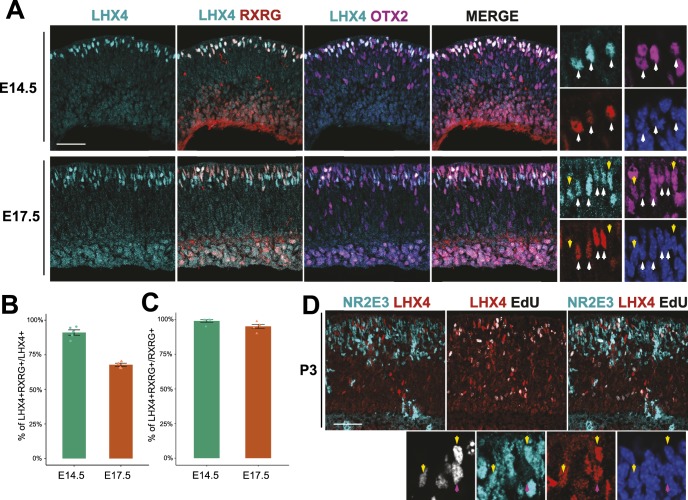
LHX4 protein is present in early photoreceptor precursors during mouse retinal development. Large: maximum-intensity projection Z-stacks; small: single planes of the same Z-stacks (A). Cross section of embryonic mouse retinas imaged for LHX4 (rabbit), RXRG, and OTX2 at the designated timepoints. Higher magnification: a single z-plane with marked LHX4 cells positive (white arrows) or negative (yellow arrows) for RXRG and OTX2. (B) Quantification of the percentage of LHX4+ cells that are also RXRG+ at E14.5 and E17.5. N = 5 retinas, one z-stack per retina. (C) Quantification of the percentage of RXRG+ cells that are also LHX4+ at E14.5 and E17.5. N = 5 retinas, one z-stack per retina. (D) Cross section of a P3 retina after a P0 EdU injection imaged for EdU, NR2E3, and LHX4 (rabbit). Higher magnification: a single z-plane with marked LHX4+/EdU+ cells positive (yellow arrows) or negative (purple arrows) for NR2E3. Scale bar represents 50 μm.

We sought to establish if this protein was also present in early photoreceptors of the mouse retina. At E14.5, using the rabbit anti-LHX4 antibody, immunoreactivity was present in the scleral portion of the retina where developing photoreceptors are located (Fig. 2A). To confirm that LHX4 was present in cone photoreceptors, we used the cone-expressed genes RXRG and OTX2 to identify these cells.^28^ RXRG is also expressed in some retinal ganglion cells but these cell types can be readily distinguished by location. Many LHX4 cells are positive for RXRG and OTX2 at E14.5, in a similar pattern to the chick retina. At E14.5, we found that 91.1 ± 1.9% (mean ± SEM) of cells positive for LHX4 were also positive for RXRG (Fig. 2B), signifying that the majority of the LHX4+ population at this timepoint were composed of early cones. In fact, nearly all cells positive for RXRG at E14.5 were also positive for LHX4 (99 ± 1%, Fig. 2C).

At a later embryonic stage (E17.5) we found that LHX4 cells still colocalized with RXRG protein, but there was an increase in LHX4+/OTX2+ cells that did not express RXRG (Fig. 2A). At this timepoint, only 67.8 ± 1.1% of LHX4+ cells were positive for RXRG, suggesting that LHX4 was active in other emerging cell populations in addition to early cones (Fig. 2B). However, 95.1 ± 1% of RXRG+ cells were positive for LHX4 at E17.5, indicating that the majority of cones retained LHX4 expression (Fig. 2C). The rabbit LHX4 polyclonal antibody had a reduced quality of staining at this timepoint, so it is possible the small fraction of cones that are not LHX4+ had undetectable LHX4 signal under these conditions or could point to a small subpopulation of cones that do not express LHX4. Additionally, we tested if LHX4 was present in postmitotic cone photoreceptors or in dividing cells. E14.5 retinas exposed to a 2-hour EdU pulse did not show a qualitative overlap between EdU and LHX4 or RXRG (Supplementary Fig. S2), which is consistent with LHX4 expression beginning after cell cycle exit. We conclude that LHX4 is a relatively specific marker for postmitotic cone photoreceptors at early stages in the mouse retina but is also present in other cell populations at later developmental stages.

### LHX4 Is Expressed in Several Developing and Adult Cell Types

Previous reports indicated that LHX4 protein was present in adult cone BCs,^29^ but little is known about its expression pattern in the developing mouse retina. We also observed strong LHX4 labeling in the upper portion of the INL in cells positive for OTX2, an adult marker for bipolar cells (Supplementary Fig. S3). Interestingly, we also noticed sparse, but positive, staining in the ONL. This LHX4 signal was located in some but not all RXRG+ cones with varying degrees of strength (Supplementary Fig. S3), suggesting that a subpopulation of cones maintain LHX4 expression.

As noted above, reports^25^,^30^ suggested that LHX4+ cells in the INL are likely developing BCs. In the mouse, our data indicated that LHX4 is initially in developing cones. However, at E17.5, RXRG+ cones no longer represent the near totality of LHX4+ cells. Since the peak of BC production is not seen until ∼P3,^31^ the identity of the remaining cells is unclear. While BCs can be produced at earlier timepoints (including E17.5), we still sought to ascertain if only LHX4+ BCs were being produced at this time, or if this LHX4 expression is present in another cell type. As rods are another OTX2+ cell type produced at this time, we aimed to see if these cells expressed LHX4 during development. Newborn mice (postnatal [P]0) were injected with EdU to mark cells undergoing S-phase at the time of injection and 3 days later the retinas were harvested. This length of time was chosen to allow some newly produced rods enough time to produce nuclear receptor subfamily 2, group E, member 3 (NR2E3), a well-known marker for rod fate.^32^ It has been previously shown that NR2E3 is also present in cone photoreceptors transiently,^33^–^35^ but, as determined previously,^36^ no cones are produced at P0. Therefore, EdU and NR2E3 colocalization should reliably mark cell types other than cones, likely rod photoreceptors. NR2E3 expression has not been reported in BCs but given the similarities in molecular profiles between BCs and photoreceptors, at this time we cannot exclude this possibility. We observed LHX4+ cells that are positive for NR2E3 and EdU at P0 (Fig. 2D). This indicates that while LHX4 is present in developing and adult BCs and cones, it is also possible that it is transiently expressed in some rod photoreceptors.

### The *LHX4::GFP* Transgenic Line Recapitulates the Endogenous LHX4 Expression Pattern

**Figure 3:**
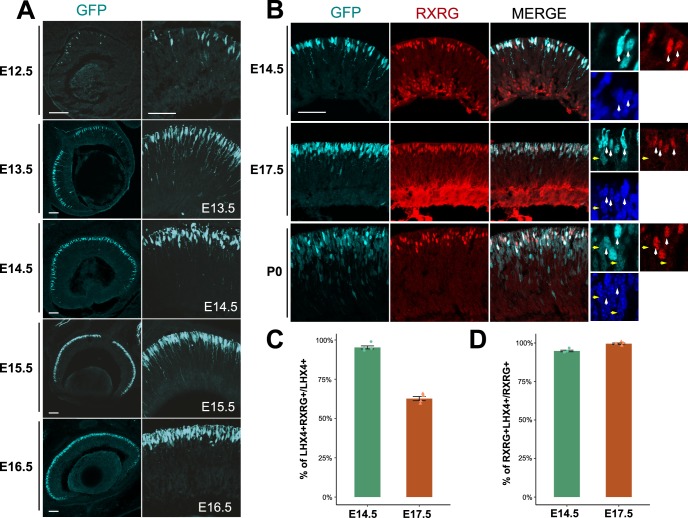
LHX4::GFP is a reliable reporter of photoreceptors during retinal development. Large: maximum-intensity projection Z-stacks; small: single planes of the same Z-stacks. Scale bar represents 50 μm. (A) Cross section along the nasal-temporal axis of embryonic LHX4::GFP mouse retinas imaged for GFP at the designated timepoints. (B) Cross section of embryonic LHX4::GFP mouse retinas imaged for GFP and RXRG at the designated timepoints. Higher magnification: a single z-plane with marked GFP cells positive (white arrows) or negative (yellow arrows) for RXRG. (C) Quantification of the percentage of GFP+ cells that are also RXRG+ at E14.5 and E17.5. N = 4 retinas, one z-stack per retina. (D) Quantification of the percentage of RXRG+ cells that are also GFP+ at E14.5 and E17.5. N = 4 retinas, one z-stack per retina.

We set out to establish if a reported *LHX4::GFP* mouse transgenic line^14^ could be a reliable tracer for early developing cones. The *LHX4::GFP* line had robust expression of GFP in the retina throughout development (Fig. 3A), and GFP signal was located in the apical portion of the retina, where photoreceptors are located during embryonic development. GFP could be detected during embryonic and postnatal development and also had stable expression in the adult retina (Supplementary Fig. S4). This line has been used previously for transcriptomic analysis,^37^ because it shows reliable activity in cone BCs. Interestingly, a subpopulation of cones is labeled by this line in the adult, and this selective expression resembles the sparse presence of LHX4 protein in the adult mouse retina. To verify if this sparse labeling was due to LHX4 presence in exclusively S or L/M types of cones, which are located in contrasting gradients in the adult retina, we assessed GFP expression in the dorsal and ventral retina.^38^ We detected no difference in GFP expression between these two areas and GFP was present in a subset of Cone Arrestin+ cells regardless of dorsal–ventral position (Supplementary Fig. S5).

We were unable to use the rabbit anti-LHX4 antibody to validate the *LHX4::GFP* expression as we encountered abnormal LHX4 immunoreactivity; not nuclear, as expected, but cytoplasmic and precisely overlapped with GFP expression. As noted in the strain description, *EGFP* was placed after the ATG of *LHX4*, replacing the *LHX4* coding region so as to function as a tracer. We amplified and sequenced the coding region downstream of the *LHX4* ATG using the reported 5′ UTR (untranslated region) primer for genotyping and, surprisingly, found it contained a 70 base-pair section of 5′ endogenous *LHX4* coding sequence immediately followed by a multiple cloning site and the *GFP* coding sequence, all in frame to produce a LHX4-GFP fusion protein. Thus, the first 34 amino acids of this fusion protein are from the sequence of LHX4 and the multiple cloning site (Supplementary Fig. S6A).

To test if this portion of LHX4 contained an epitope for the LHX4 antibody, we amplified the coding sequence from the *LHX4::GFP* genomic DNA, cloned it into a misexpression vector, and electroporated it into the chick retina (Supplementary Figs. S6B–D). Control retinas had no detectable GFP and normal staining of LHX4 with the rabbit antibody (Supplementary Fig. S6B). In contrast, cells electroporated with the CAG::5pLHX4GFP construct were strongly immunoreactive to the LHX4 antibody in a pattern that completely overlapped that of the cytoplasmic GFP signal (Supplementary Fig. S6C). The rabbit-LHX4 still detected endogenous LHX4, as we imaged the same retinas at the edge of the electroporation patch with higher gain and observed endogenous protein expression alongside overexposed electroporation signal (Supplementary Fig. S6D).

Therefore, to assess if GFP recapitulates LHX4 expression patterns and its activity in early cones, we resorted to examination of RXRG expression in GFP+ cells at two relevant timepoints of embryonic development. At E14.5, the GFP reporter faithfully recapitulated LHX4 expression as 95.3 ± 1% of all GFP+ cells were positive for RXRG (Fig. 3C). Likewise, at E17.5 only 65.7 ± 1.3% of GFP cells were RXRG+. As the LHX4 immunodetection showed, nearly all RXRG+ cones are positive for LHX4 at both E14.5 and E17.5. This was also true in the LHX4::GFP+ population, where 94.8 ± 0.5% and 99.5 ± 0.5% of all RXRG cells, respectively, were positive for the GFP reporter (Fig. 3D). Taken together, these data suggest that the *LHX4::GFP* BAC reporter reliably recapitulates LHX4 protein expression and is a dependable marker for cone photoreceptors during early retinal development.

### Single-Cell Sequencing of *LHX4::GFP* Cells in the E14.5 Developing Mouse Retina

**Figure 4:**
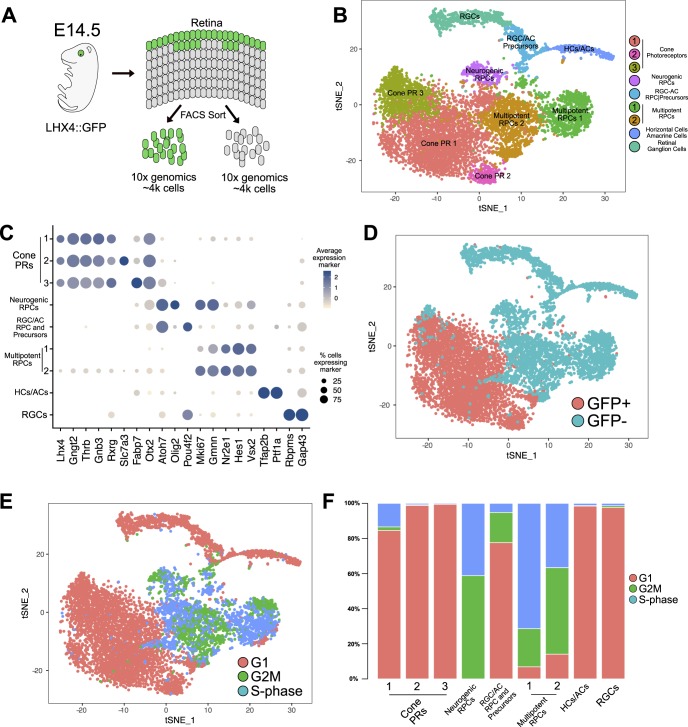
TSNE: single-cell sequencing of LHX4::GFP E14.5 developing retina. (A) Schematic of experimental design for single-cell collection and sequencing. (B) TSNE plot of all GFP+ and GFP− cells collected, analyzed in tandem, and displaying the results of unsupervised cluster analysis with the assigned cell class according to their molecular signature. (C) Dot plot displaying the average expression and percentage of cells expressing specific markers in each cluster. Markers displayed on the x-axis were used for assignment of cell class to each cluster. (D) Same TSNE plot as (B) displaying the original source of each cell, GFP+ or GFP− samples. (E) Same TSNE plot as (B) displaying the cell cycle assignment of each cell. (F) Percentage of cells in each cluster assigned to different cell cycle phases.

Having verified that the *LHX4::GFP* reporter is a marker for cone photoreceptors in the early stages of mouse retinal development, we took advantage of this system to examine the molecular profile of early cone photoreceptors using single-cell RNA sequencing. *LHX4::GFP* E14.5 littermates were screened for GFP expression and positive retinas were pooled and dissociated in preparation for FACS sorting (Fig. 4A). GFP+ and GFP− cells were collected, and approximately 4000 cells were sequenced per condition using the 10× Chromium platform.

Using the Seurat program,^19^ we performed an unsupervised clustering analysis on the combined cell transcriptomes from both conditions that passed standard 10× QC (GFP+: 3728, GFP−: 4444). TSNE projections revealed that the GFP+ and GFP− populations clearly segregated, consistent with *LHX4::GFP* cells being a molecularly distinct population (Fig. 4B). The analysis separated the cells into nine distinct clusters, which were identified by established markers and bore hallmarks of known cell classes in the developing retina (Figs. 4C, 4D; Supplementary File S1).

Two cell clusters were assigned as multipotent RPCs based on expression of multipotent RPC markers such as *VSX2*, *HES1*, and *NR2E1*, among others, as well as a large percentage of cycling cells (Figs. 4E, 4F).^20^ One other cluster was predominantly composed of cycling cells, with markers such as *OLIG2*, *NEUROG2*, *OTX2*, and *ATOH7*, which identify these as likely neurogenic RPCs^39^,^40^ with limited mitotic potential. A second cluster had a smaller fraction of cycling cells, high *ATOH7* levels, and expression of *POU4F2*, but no *OTX2* or *OLIG2*, which we assigned to retinal ganglion cell (RGC)/amacrine (AC) RPCs and precursors as they exhibited markers of likely differentiation to RGCs or ACs.

Of the cell clusters assigned as mostly postmitotic, one was assigned as RGCs as it displayed known markers *RBPMS* and *GAP43*.^41^,^42^ Another assigned to AC/HCs, exhibiting markers for both these fates, like *TFAP2B* and *PTF1A*.^43^,^44^ As previously reported,^40^ while ACs and HCs are distinct fates, it is difficult to separate them by their transcriptomes in early development and without an appropriate amount of sequenced cells for proper resolution.

Three clusters were assigned as cone photoreceptors. As expected from our previous data, these consisted of the near totality of GFP+ cells and displayed markers of cone photoreceptors: *THRB*, *RXRG*, *GNGT2*, and *GNB3* (Figs. 4C, 4D), as well as *LHX4*. We did detect some sparse *NRL*-positive cells within this population, possibly reflecting some activity of the *LHX4* reporter in rod photoreceptors. The cell cycle phase of these clusters is consistent with our previous data suggesting LHX4 is in postmitotic cones at E14.5. As a result of the sorting strategy, cone representation was high, which allowed the clustering analysis to resolve cone subpopulations. All cone clusters had high levels of established markers, but two subpopulations differed in expression of genes. The highest differential marker for one of the populations was *FABP7*, a previously characterized marker for cones in the adult murine retina^45^ with reported expression in the developing retina^46^ but not at this early stage. Meanwhile, a second subpopulation had increased levels of solute-carrier genes like *SLC7A3* and *SLC7A5* (Fig. 4C). Our analysis indicates that we successfully sorted and sequenced a developing E14.5 retina and identified its cell populations while enriching for cone photoreceptors.

### Early Cone Marker Identification in *LHX4::GFP* Cells

**Figure 5:**
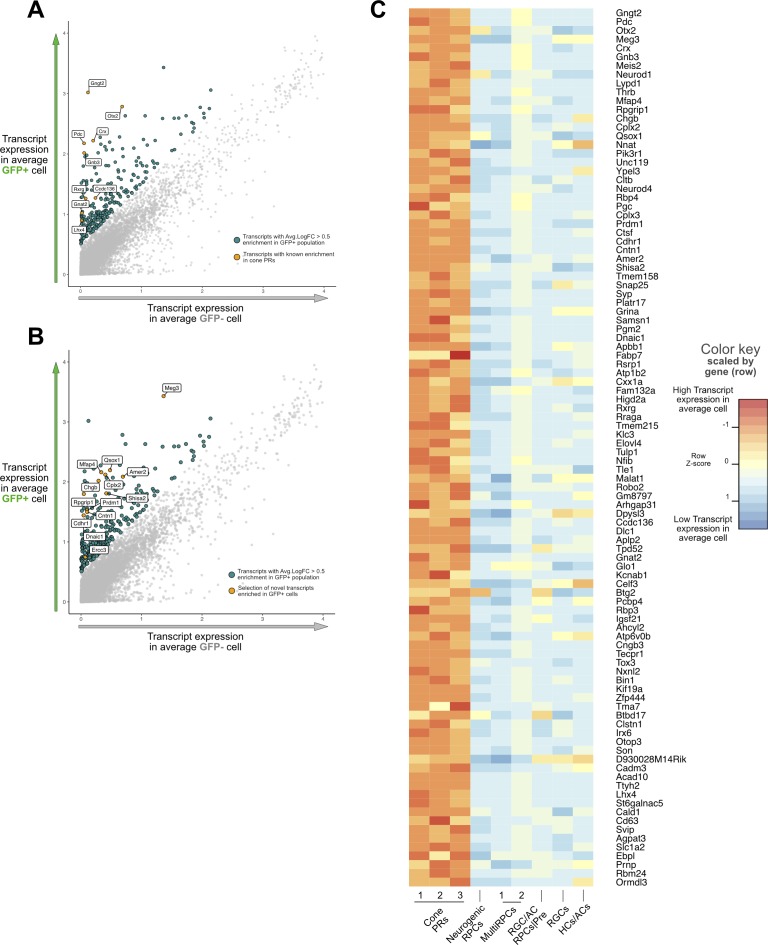
Identification of known and novel transcripts enriched in cone photoreceptors in LHX4::GFP+ cells. (A) Plot displaying the expected transcript expression in an average GFP+ (y-axis) or GFP− (x-axis) cell. Highlighted are all genes with >0.5-fold change enrichment in GFP+ cells, and labeled are a selection of cone-related transcripts. (B) Same plot as (A) labeling novel transcripts enriched in the same population. (C) Heatmap of top 100 transcripts enriched in GFP+ cells. Color indicates the expected transcript expression of that gene in an average cell for each cluster identified. Expression values are scaled per gene.

We sought to use this dataset to find new markers for early cone photoreceptors. Using Seurat, we performed a differential expression analysis, comparing the GFP+ population, which we established as cones, with the GFP− population, which should encompass the other concurrent populations in the developing retina. Additionally, for visualization purposes and as a proxy for average population levels of transcript expression, we calculated the expression of an average GFP+ and a GFP− cell and used this in combination with the differential expression results. We identified over 898 significantly differentially expressed transcripts with enrichment in cones. As expected, we identified known cone markers as highly enriched in the GFP+ population (Fig. 5A; Supplementary File S2). We then looked for novel transcripts enriched in this population (Fig. 5B). A heatmap for the top 100 cone-enriched transcripts with the transcript expression of an average cell in every individual cluster is included in Figure 5C.

The above analysis identified genes enriched in early cone photoreceptors compared to the other cell types present at that time. We first compared these results to other studies that have reported cone transcriptome analyses in the mouse or human (Supplementary Figs. S7A, S7B). Only one study has reported a transcriptome from early developing cones, similar to this one,^4^ through the use of human fetal explants infected with cone opsin promoter reporter viruses, determining early and late phases of gene expression in labeled versus nonlabeled cells. A number of genes are enriched in both datasets (165 genes in early and 211 in late fetal cones), suggesting that the mouse is a potential model for investigating the function of these genes in cone photoreceptors. (Supplementary Fig. S7; Supplementary File S3, S4).

Additional datasets of purified adult rods and cones from the mouse have been used to identify differential transcripts between these two types of photoreceptors.^7^ Comparison to this dataset identified that 251 of the genes in our dataset were enriched in adult cones and 121 were enriched in adult rods (Supplementary Fig. S7; Supplementary File S3, S5). Adult cone-enriched genes present in this dataset support the cell specificity of known early markers detected in our dataset, such as *RXRG*, as well as many genes not explored in detail before, such as *QSOX1* or *LHX4*.

To corroborate the expression of some of these genes as enriched in early cones we cross-referenced the Transcripts Per Million (TPM) values of these genes determined by a previous transcriptome analysis of E14.5 mouse retina (Supplementary File S6).^47^ Across all cell clusters, only five genes were reported to have 0 TPMs in the previous analysis, though none of these genes were in the cone genes identified here. We observed that many of the novel cone genes have low TPMs, which could have contributed to the fact that they have not been identified previously in cone cells. To determine if the expression pattern of some of the identified cone genes was consistent with cone photoreceptor location, we examined a dataset of E15.5 RNA in situ hybridization data generated by the Allen Brain Atlas. We identified 49 genes as having been tested in the Allen Brain Atlas out of the 100 most enriched genes of each of the three identified cone clusters (Supplementary File S6). Of these genes, 33% gave no signal, suggesting that the probe was not sensitive enough to detect them or their expression had diminished by E15.5; 45% gave a detectable photoreceptor pattern along the scleral edge of the retina, and 22% gave a detectable RNA in situ signal that did not correspond to an obvious photoreceptor signal. Most of the genes in this last category were specific to cone clusters 2 and 3. Several examples from this analysis of genes with a photoreceptor-like photoreceptor pattern are shown in Supplementary Figure S8.

**Figure 6:**
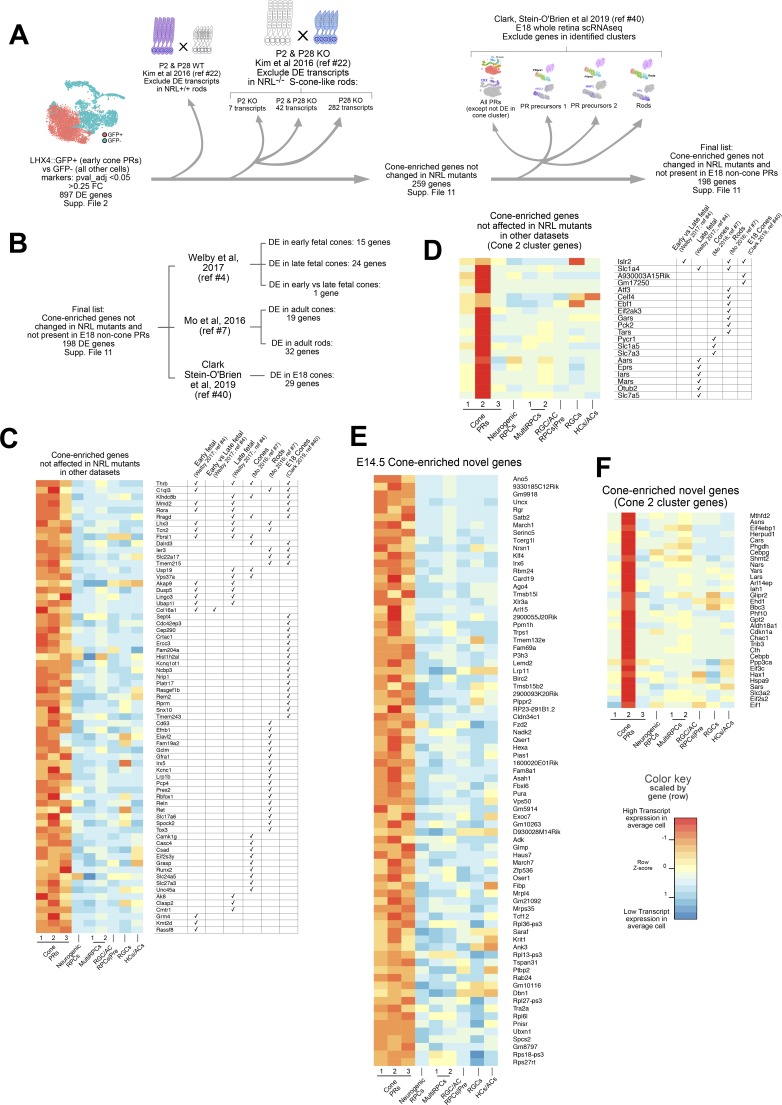
E14.5 genes not changed in NRL mutants and novel cone-enriched genes. (A) Schematic describing the comparison and selection of genes enriched in E14.5 cones that are not changed in the NRL mutant dataset and not present in early rods or photoreceptor precursors at E18.^41^ DE, differentially expressed. (B) Number of genes found overlapping in the displayed datasets from the final list of genes. (C) Heatmap of transcripts enriched in E14.5 cones that are not changed in the NRL mutant dataset and not present in early rods or photoreceptor precursors at E18. The genes displayed are also present in the datasets analyzed, and their presence is indicated by a checkmark in the right table. Color indicates the expected transcript expression of that gene in an average cell for each cluster identified. Expression values are scaled per gene. (D) Same as (C) but for genes found in the cone 2 cluster. (E) Heatmap of novel genes found in this study to be enriched in E14.5 cones but not present in any of the other datasets. Color indicates the expected transcript expression of that gene in an average cell for each cluster identified. Expression values are scaled per gene. (F) Same as (E) but for novel genes in the cone 2 cluster.

We next were interested in determining whether the genes associated with early cone genesis were negatively regulated by the rod photoreceptor factor *NRL*. A prevalent model for *NRL* function is that it serves as a fate switch in photoreceptor precursors, with *NRL*-negative precursors becoming cones and *NRL*-positive ones becoming rods.^8^,^12^,^48^ In *NRL* mutants, the photoreceptors that normally would become rods are found to undergo a morphologic and gene expression change that could suggest a rod-to-cone fate switch. However, it has been noted that these cells are morphologically distinguishable from normal cones; and, in addition, known early cone genes, such as *THRB* and *RXRG*, are unaltered in newborn photoreceptors, which suggests that *NRL* may not be a master regulator of this fate choice.^8^,^12^,^49^ As there are few known early cone genes, we sought to identify other cone photoreceptor genes, in addition to *THRB* and *RXRG*, that are regulated independently of *NRL*. We first identified those genes in our dataset that were dysregulated either positively or negatively in *NRL* knockout photoreceptors (Supplementary File S7). The *NRL* dataset used contained multiple isoforms of genes, but to apply a stringent criterion, any isoform that showed dysregulation at either P2 or P28 in *NRL* mutants led to that gene being removed, leaving a total of 259 *NRL*-independent genes (Fig. 6A). Interestingly, many of the cone-enriched genes present at E14.5 are only altered in *NRL* mutants at P28 and not also at P2 when a large number of rods would be generated (see Discussion).

As some of these genes likely represent pan-photoreceptor genes that would not be expected to be under the transcriptional control of *NRL*, we identified those genes also expressed in developing rods. As an identifiable rod cluster was not present in our E14.5 dataset, we analyzed a recently generated single-cell dataset from E18 whole retinas.^40^ The data from two replicates of E18 were extracted and we performed an unbiased clustering analysis using Seurat (Supplementary Fig. S9).^19^ The detected nine clusters were assigned to known cell types through marker expression. Within those, two clusters were positive for *CRX* and, thus, determined to be likely photoreceptors. First, all differentially expressed transcripts for photoreceptors at E18 were determined (Supplementary File S8; Supplementary Fig. S8) and then these cells were reanalyzed for further subclustering. We were able to determine a cluster with cone signature, rod signature, and two precursor clusters. From those, we determined all differentially expressed transcripts between photoreceptor clusters (Supplementary File S9; Supplementary Fig. S8). Interestingly, many of the genes detected as enriched in early cone photoreceptors in this study have been identified as differentially expressed across retinal pseudo-time by Clark et al.^40^ (Supplementary File S10).

Removal of genes with expression in any of the non-cone clusters at E18 produced 198 *NRL*-independent cone-enriched genes (Figs. 6A, 6B; Supplementary File S11). To provide some measure of confirmation of the cone/photoreceptor-associated expression of these genes, we identified those genes with cone-enriched expression or specifically enriched in cone cluster 2 that were also identified in the previously mentioned datasets (Figs. 6C, 6D; Supplementary File S11). This left a set of genes with cone-enriched expression or specifically enriched in cone cluster 2 that have been identified to date only in this study as cone associated (Figs. 6E, 6F).

## Discussion

Our current knowledge of the molecular and cellular events involved in cone photoreceptor development is incomplete. One missing dataset has been a comprehensive gene expression analysis of endogenously developing cones. Without such knowledge, the rational design of strategies to induce cone formation and adequate benchmarks to assess these cones, such as from stem cell cultures, are not possible. Here we identify *LHX4* as a novel and highly specific marker of cone photoreceptors in the early stages of their development and the *LHX4::GFP* reporter mouse as a tool to detect and analyze these cells in the developing mouse retina.

The single-cell transcriptome analysis allowed for an unprecedented molecular examination of early cone photoreceptors. With the increased representation that the *LHX4::GFP* mouse permitted for the purification of this relatively rare cell type, we were able to identify subclusters of cones at this timepoint. The biological significance of these subclusters, however, remains, to be determined. They could reflect temporal differences in the differentiation process or spatial effects linked to the dorsal–ventral position of cells, which is known to influence cone opsin expression at later timepoints. One distinct cluster upregulates the *SLC7A3* and *SLC7A5* genes. SLC7A5 is a known thyroid hormone transporter for T3/T4 states^50^ that has been reported in human fetal retinas and organoids.^51^,^52^ Because these transcriptomic analyses were done in whole retinas it was not clear if this expression was specific to any particular cell population, but our data suggest that mouse cone photoreceptors specifically express these genes. *SLC7A3* has not been reported in the retina but likely plays a similar role, as its expression has been linked to T3 administration in the brain.^53^ Thyroid hormone and its targets are known to affect M-cone differentiation.^15^,^51^,^54^–^56^ We found several other thyroid-related genes throughout the retina, but only *THRB* was exclusive to cones at this time. For example, there was almost no detectable amount of *DIO2* in any cell but many more counts of *DIO3*, although these were present in the multipotent RPC clusters. Another observation of cone heterogeneity was in the expression of LHX4 protein and the *LHX4::GFP* reporter in more mature retinal tissue. Whether this sustained expression of LHX4 in subsets of cones is biologically relevant or is an epigenetic or temporal phenomenon without a functional significance warrants further study.

There is a major interest in understanding the molecular differences between cone and rod photoreceptors and how these differences arise during development. This would provide insights into how these two classes of photoreceptors evolved as well as inform the strategic development of methods to specifically produce these cell types. The predominant photoreceptor determination framework is that cones and rods developmentally diverge based solely on whether newborn photoreceptors begin to express the *NRL* TF or not. The analysis performed here with multiple previously published datasets and the current one suggests that endogenous newborn cones have a unique molecular signature compared to the induced cones that form in the *NRL* knockout. There are caveats that must be considered. For one, the current study used a massive enrichment of cones, which allowed for increased power to detect gene expression differences between cones and other cells. Comparison to other datasets that had less statistical power may have led to the false conclusion that a gene was not differentially expressed. In addition, we compared the profile of embryonic cones to *NRL*-dysregulated genes at P2. There could be currently unidentified signaling factors that are temporally different and influence gene expression that could account for these differences. It is important to note that a large number of cone genes are in fact dysregulated in the *NRL* mutant. However, it is interesting that many of these genes found in early cones, such as *RXRG*, are not dysregulated in early rods, but are only significantly changed in much more mature rods. What the cause of this delay is and whether it impacts the differentiation of these transformed cells to a more bona fide cone photoreceptor signature is not known. In any case, these differences warrant further examination of the utility and validity of using photoreceptors from the *NRL* knockout model as a substitute for naturally formed cones.

## Supplementary Material

Supplementary Figure S1

Supplementary Figure S2

Supplementary Figure S3

Supplementary Figure S4

Supplementary Figure S5

Supplementary Figure S6

Supplementary Figure S7

Supplementary Figure S8

Supplementary Figure S9

Supplementary File S1

Supplementary File S2

Supplementary File S3

Supplementary File S4

Supplementary File S5

Supplementary File S6

Supplementary File S7

Supplementary File S8

Supplementary File S9

Supplementary File S10

Supplementary File S11
