# Supplementary material for: Identification of Genes With Enriched Expression in Early Developing Mouse Cone Photoreceptors
Source: Invest Ophthalmol Vis Sci. 2019 Jul;60(8):2787–99. doi: 10.1167/iovs.19-26951 (PMC6607928; doi:10.1167/iovs.19-26951)
Supplement: Supplementary Figure S1 [file iovs-60-07-32_fig_S1.pdf]

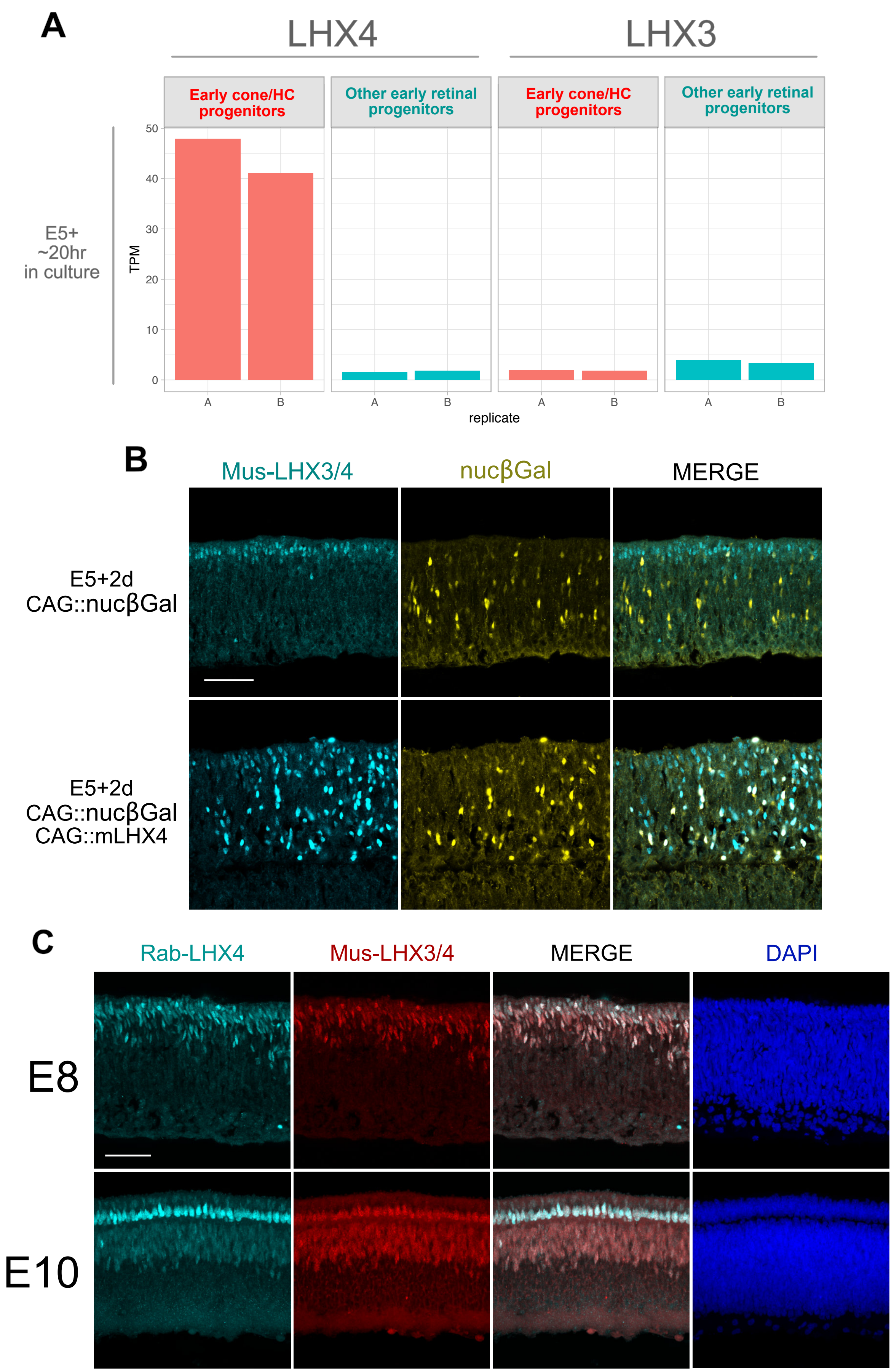

Supplemental Figure 1

Supplemental Figure 1 - LHX4 and not LHX3 is detected in early chick retina development.

(A) TPM values for LHX4 and LHX3 in Cone/HC progenitors and other early retinal progenitors.

(B) Cross-section of a chicken retina electroporated at E5 and cultured for ~20hrs. Electroporated constructs are denoted on the left side. Retinas were imaged for βGal and LHX3/4 with mouse anti-LHX3 (DSHB).

(C) Maximum intensity projections of cross-section in chicken retinas at designated timepoints imaged for LHX4 with mouse (DSHB) and rabbit (Proteintech) antibodies. Scale bar represents 50 μm.
