# Supplementary material for: Identification of Genes With Enriched Expression in Early Developing Mouse Cone Photoreceptors
Source: Invest Ophthalmol Vis Sci. 2019 Jul;60(8):2787–99. doi: 10.1167/iovs.19-26951 (PMC6607928; doi:10.1167/iovs.19-26951)
Supplement: Supplementary Figure S2 [file iovs-60-07-32_fig_S2.pdf]

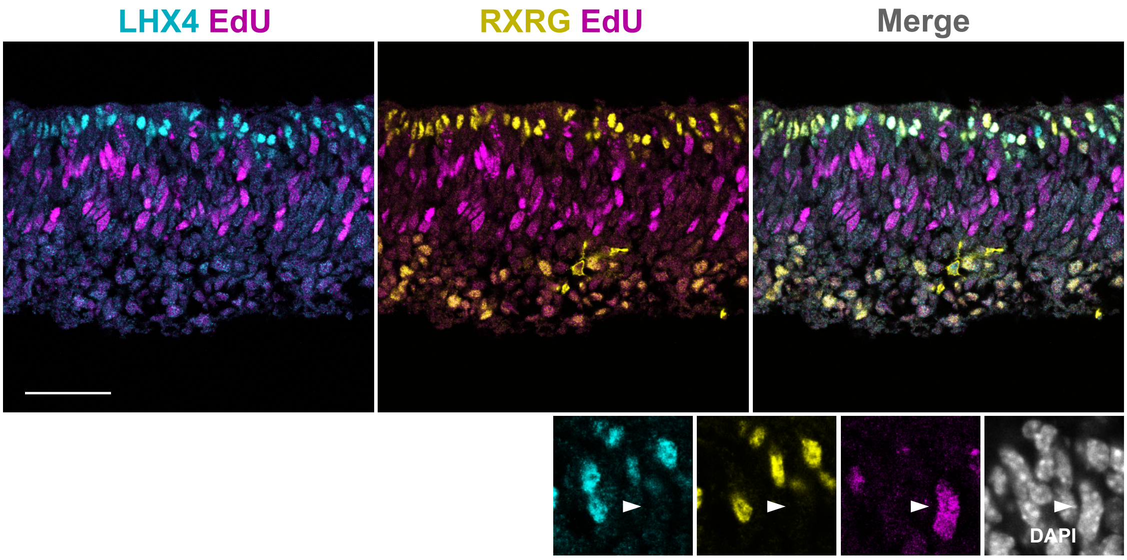

Supplemental Figure 2

Supplemental Figure 2 - LHX4 expression starts in post-mitotic cones

Cross-section of a E14.5 mouse retina, developed for EdU (2hr pulse) and imaged for LHX4 and RXRG. Small panels are digitally zoomed. Arrow points at representative EdU cell negative for LHX4 and RXRG. Scale bar represents 50 μm.
