# Supplementary material for: Identification of Genes With Enriched Expression in Early Developing Mouse Cone Photoreceptors
Source: Invest Ophthalmol Vis Sci. 2019 Jul;60(8):2787–99. doi: 10.1167/iovs.19-26951 (PMC6607928; doi:10.1167/iovs.19-26951)
Supplement: Supplementary Figure S3 [file iovs-60-07-32_fig_S3.pdf]

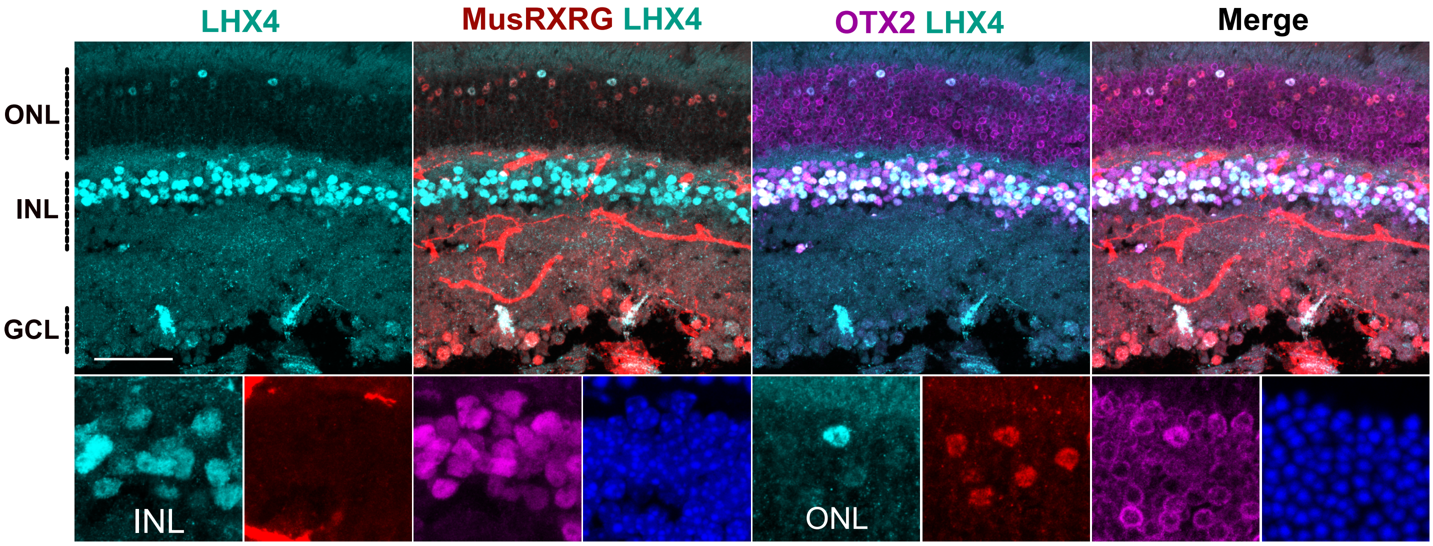

Supplemental Figure 3

Supplemental Figure 3 - LHX4 is expressed in cone bipolar cells and a subpopulation of cones in the adult mouse retina.

Cross-section of a P27 mouse retina imaged for LHX4, RXRG, and OTX2. Higher magnification panels in the ONL and INL as specified. Large panels are maximum intensity projection Z-stack and small panels are single planes of the same Z-stack. Scale bar represents 50 µm.
