# Supplementary material for: Identification of Genes With Enriched Expression in Early Developing Mouse Cone Photoreceptors
Source: Invest Ophthalmol Vis Sci. 2019 Jul;60(8):2787–99. doi: 10.1167/iovs.19-26951 (PMC6607928; doi:10.1167/iovs.19-26951)
Supplement: Supplementary Figure S4 [file iovs-60-07-32_fig_S4.pdf]

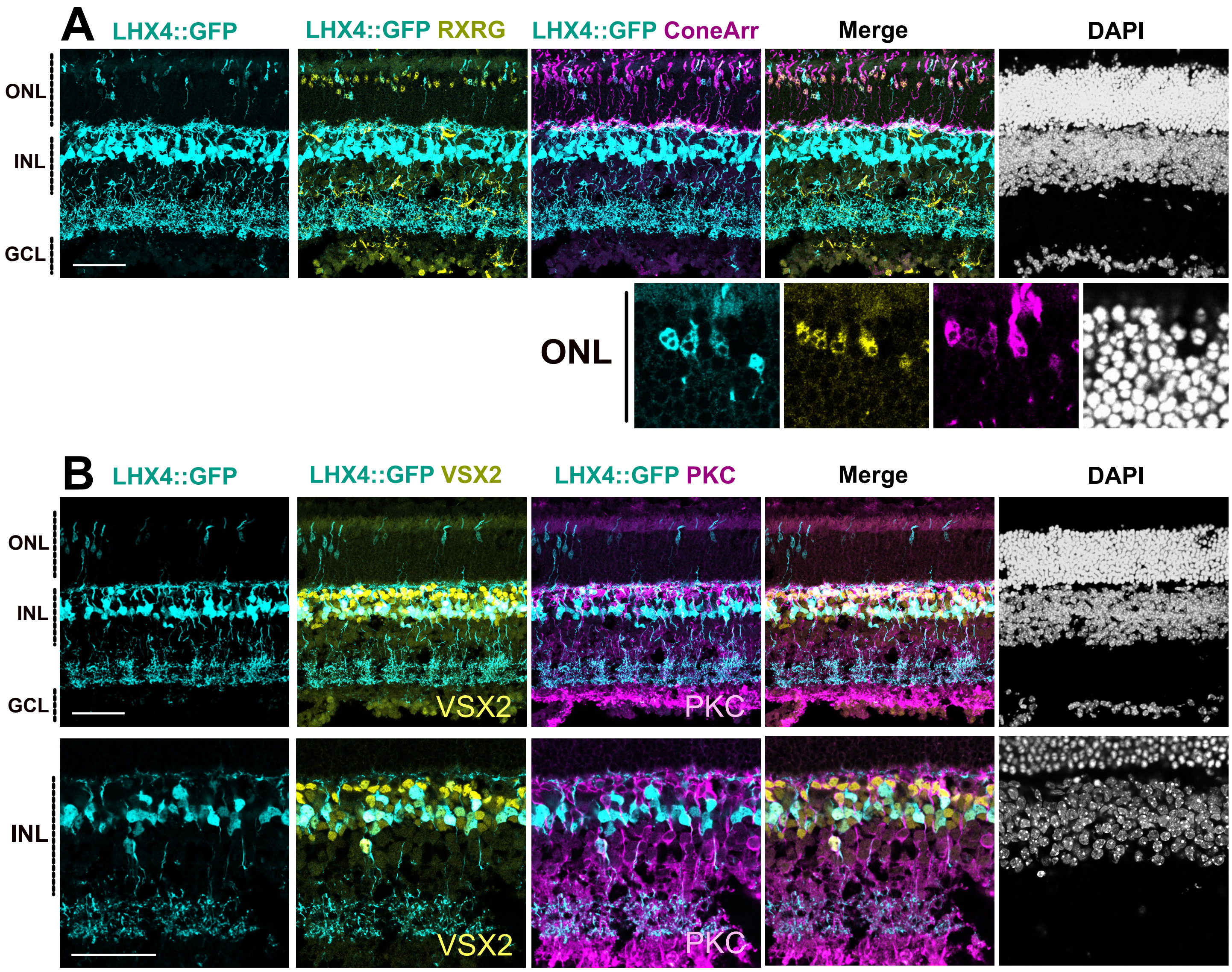

Supplemental Figure 4

Supplemental Figure 4 - LHX4::GFP reporter is active in cone bipolar cells and a subset of cones in the adult mouse retina.

Cross-section of P27 mouse retinas imaged for GFP, RXRG, and Cone Arrestin in (A), and GFP, VSX2 and PKC in (B). Higher magnification panels in (A) show a single z-plane in the ONL. Large panels are maximum intensity projections Z-stacks and small panels are single planes of the same Z-stacks. Scale bar represents 50 µm.
