# Supplementary material for: Identification of Genes With Enriched Expression in Early Developing Mouse Cone Photoreceptors
Source: Invest Ophthalmol Vis Sci. 2019 Jul;60(8):2787–99. doi: 10.1167/iovs.19-26951 (PMC6607928; doi:10.1167/iovs.19-26951)
Supplement: Supplementary Figure S5 [file iovs-60-07-32_fig_S5.pdf]

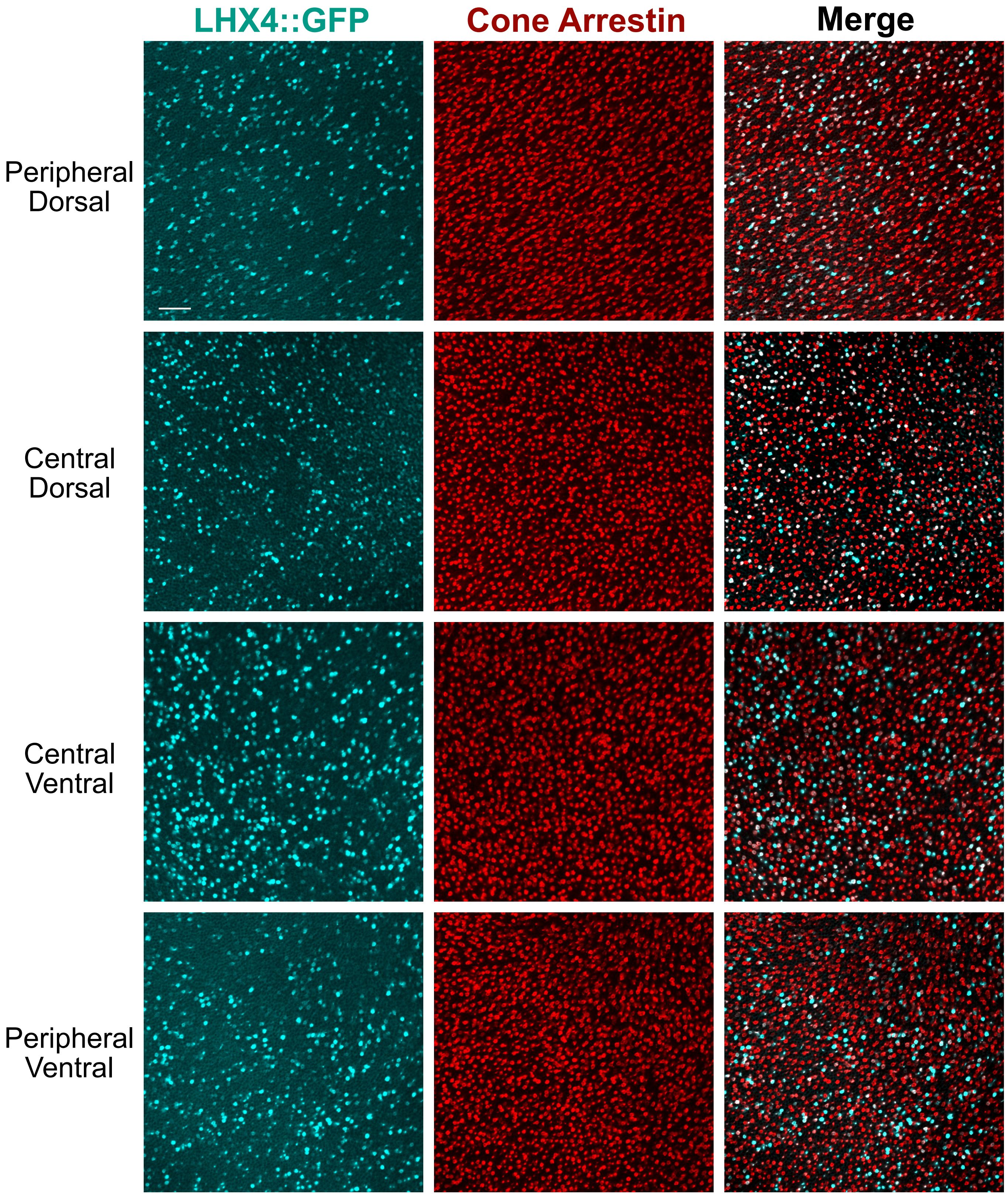

Supplemental Figure 5

Supplemental Figure 5 - LHX4::GFP reporter in the adult is active but not subject to dorsal-ventral gradient.

Whole mount of a P27 LHX4::GFP mouse retina imaged for GFP and Cone Arrestin imaged at the ONL. Scale bar represents 50 μm.
