# Supplementary material for: Identification of Genes With Enriched Expression in Early Developing Mouse Cone Photoreceptors
Source: Invest Ophthalmol Vis Sci. 2019 Jul;60(8):2787–99. doi: 10.1167/iovs.19-26951 (PMC6607928; doi:10.1167/iovs.19-26951)
Supplement: Supplementary Figure S6 [file iovs-60-07-32_fig_S6.pdf]

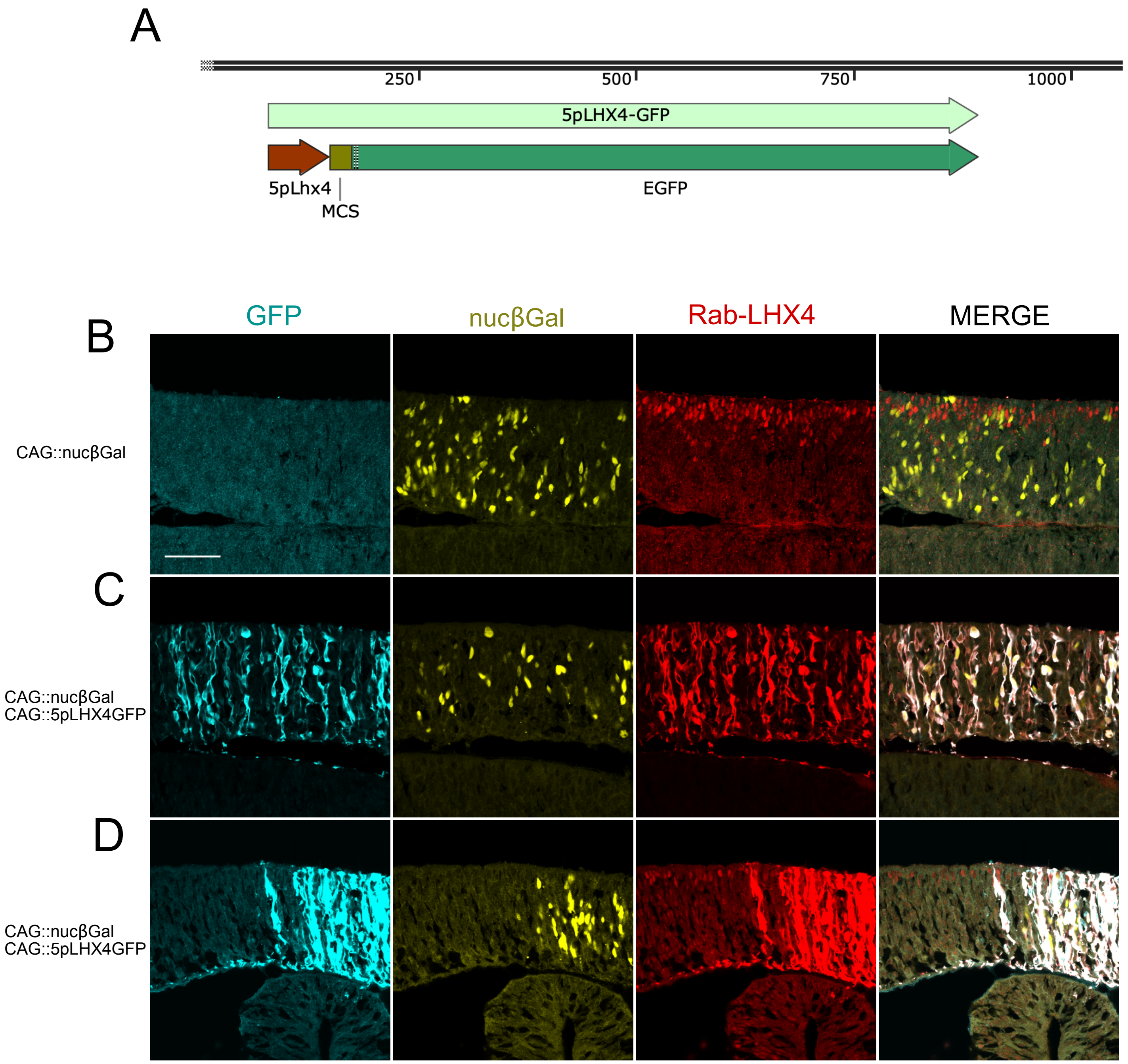

Supplemental Figure 6

Supplemental Figure 6 - LHX4::GFP transgene retains part of the LHX4 5' coding sequence and produces a N-terminusLHX4-GFP fusion.

(A) Schematic of coding region amplified from LHX4::GFP locus containing a portion of the N-terminus of LHX4. Black bars represent DNA length. MCS is multiple cloning sequence. Top light green bar represents predicted fusion protein.

(B-D) Cross-section of chicken retinas after electroporation and ~20hr incubation. Retinas are imaged for GFP, RbLHX4 and βGal. In the case of (C), imaged at the edge of the electroporated area with increased exposure to show endogenous LHX4 signal. Scale bar represents 50 μm.
