# Supplementary material for: Identification of Genes With Enriched Expression in Early Developing Mouse Cone Photoreceptors
Source: Invest Ophthalmol Vis Sci. 2019 Jul;60(8):2787–99. doi: 10.1167/iovs.19-26951 (PMC6607928; doi:10.1167/iovs.19-26951)
Supplement: Supplementary Figure S7 [file iovs-60-07-32_fig_S7.pdf]

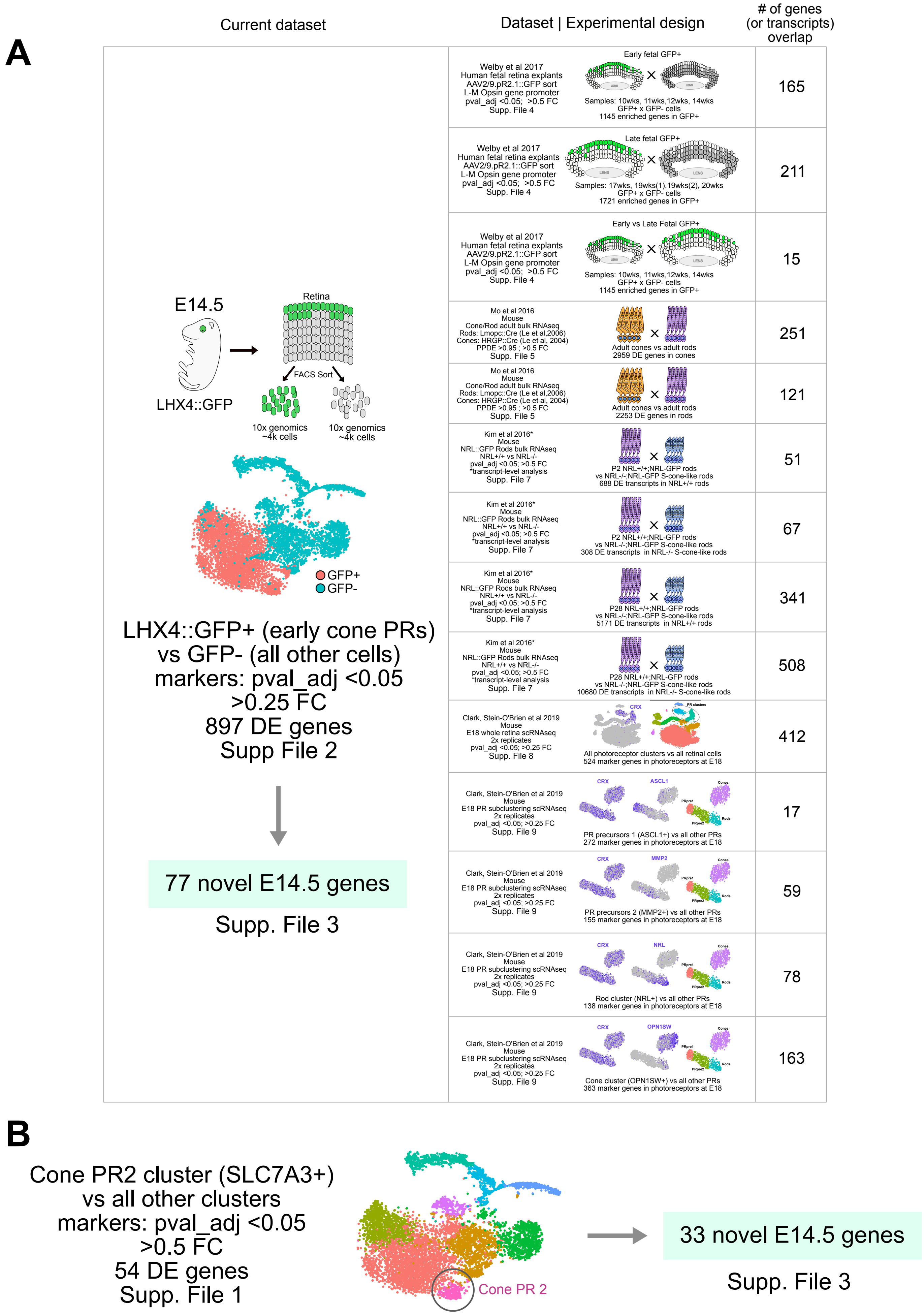

Supplemental Figure 7

Supplemental Figure 7 - Summary of datasets compared with E14.5 cone-enriched genes from LHX4-GFP current dataset.

A) Table describing the datasets used for comparison and the number of overlapping genes with E14.5 cone-enriched genes. For each report (Welby, Mo, Kim and Clark), datasets are divided in subsections depending on enrichment in a particular cell type, as denoted. DE=Differentially Expressed

B) Summary of comparison with Cone 2 cluster enriched genes.
