# Supplementary material for: Identification of Genes With Enriched Expression in Early Developing Mouse Cone Photoreceptors
Source: Invest Ophthalmol Vis Sci. 2019 Jul;60(8):2787–99. doi: 10.1167/iovs.19-26951 (PMC6607928; doi:10.1167/iovs.19-26951)
Supplement: Supplementary Figure S8 [file iovs-60-07-32_fig_S8.pdf]

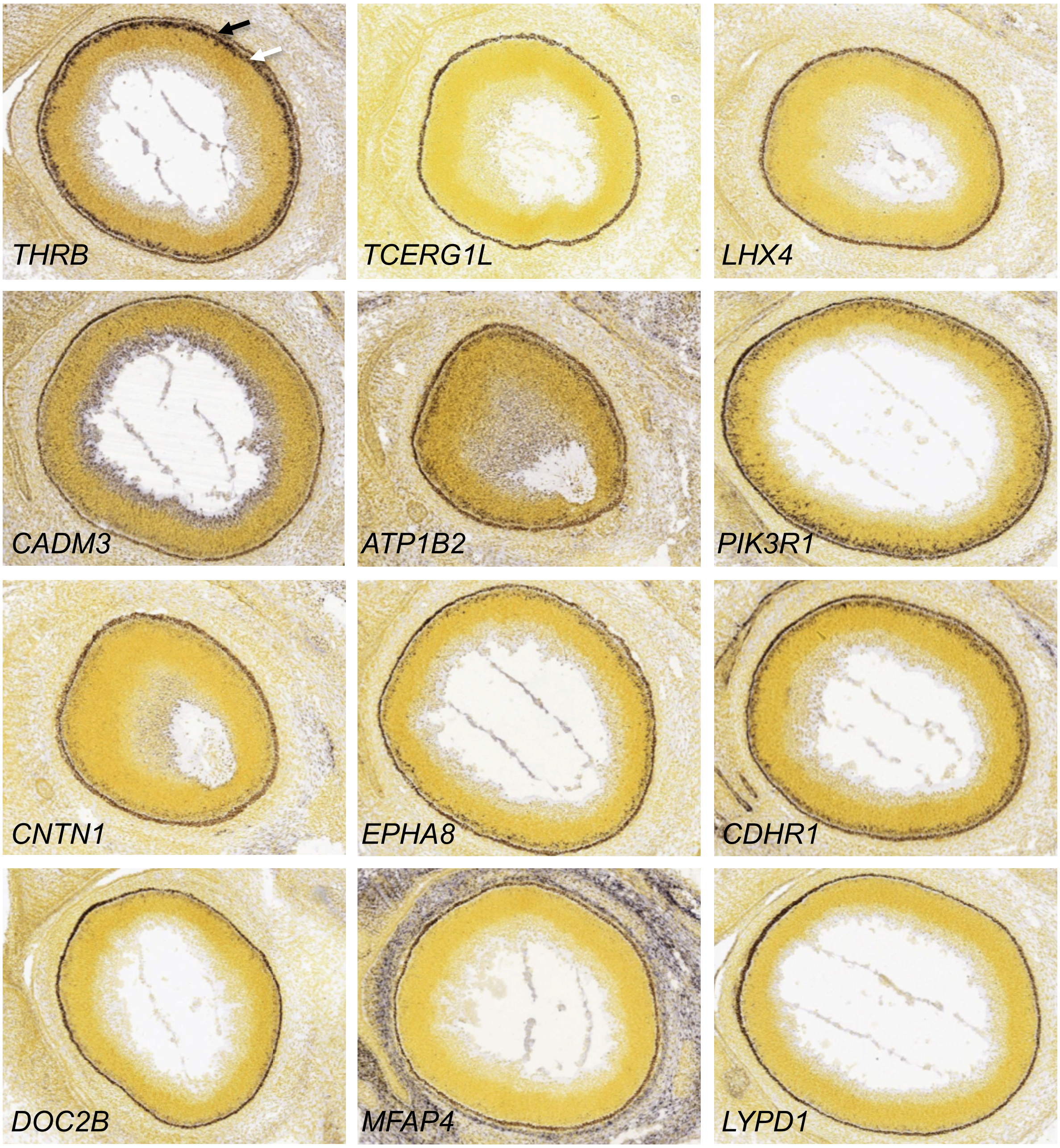

Supplemental Figure 8

Supplemental Figure 8 - RNA in situ hybridization images of a subset of cone-enriched genes. Sagittal images of E15.5 mouse embryos (front of embryo to the left) centered on the retina region and hybridized with probes to the gene shown in the bottom left of the image. All images were obtained from and are credited to the Allen Institute website at www.alleninstitute.org and specific links to the image for each gene are found in Supplemental File 11. Arrows in the image for the previously described cone gene *THRB* were added to show the location of the retinal pigmented epithelium (black arrow) and the location of cone photoreceptors (white arrow). A photoreceptor pattern was not observed for the TCERG1L probe and serves as a point of comparison to the other images.
