# Supplementary material for: Identification of Genes With Enriched Expression in Early Developing Mouse Cone Photoreceptors
Source: Invest Ophthalmol Vis Sci. 2019 Jul;60(8):2787–99. doi: 10.1167/iovs.19-26951 (PMC6607928; doi:10.1167/iovs.19-26951)
Supplement: Supplementary Figure S9 [file iovs-60-07-32_fig_S9.pdf]

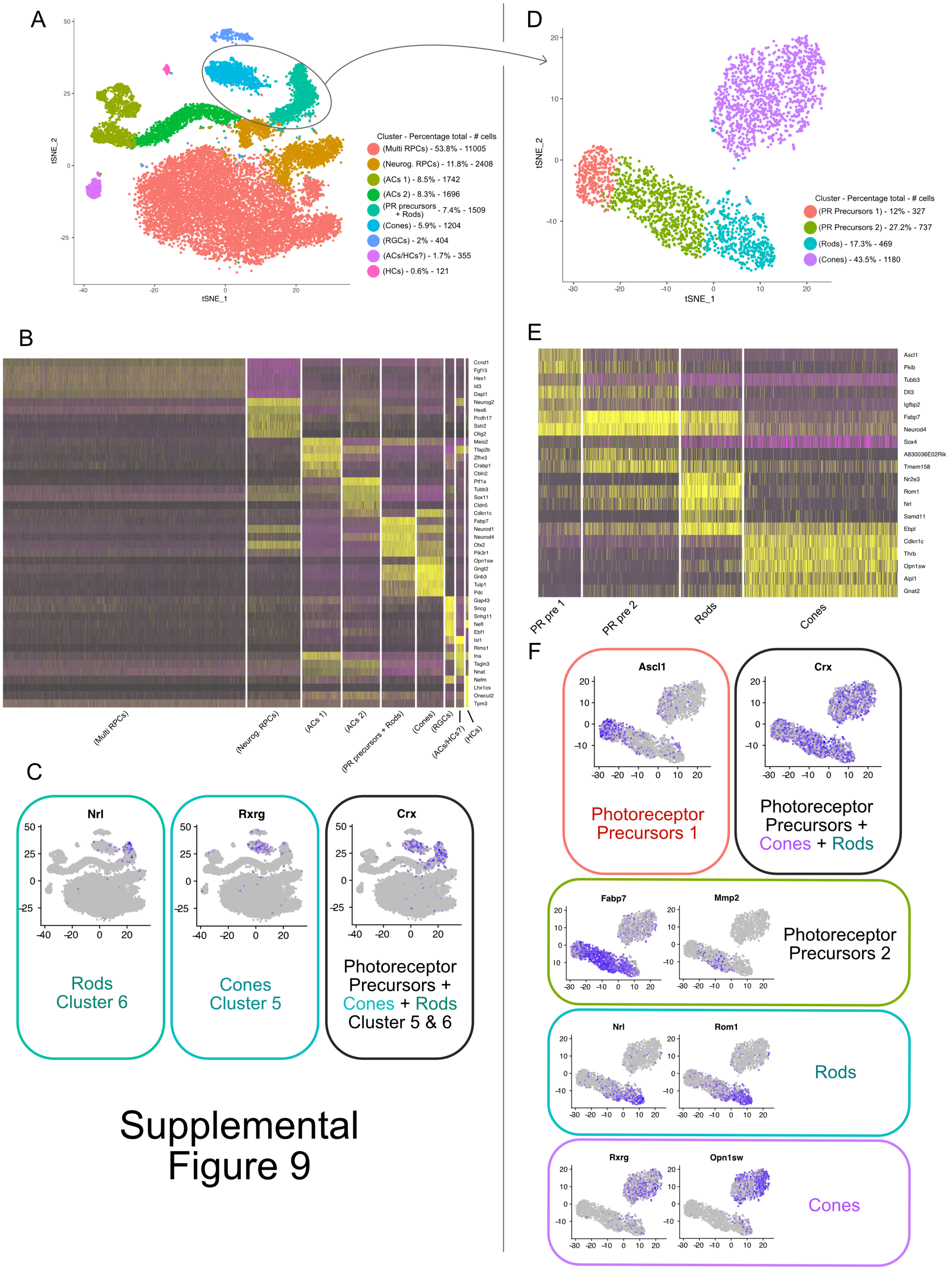

Supplemental Figure 9

Supplemental Figure 9 – Unsupervised clustering analysis of E18 timepoint from Clark & O'Brien et al 2019

A) TSNE plot of two replicates of E18 whole mouse retinas. Clusters are assigned according to markers shown in B and C. Highlighted are the photoreceptor clusters selected for further subclustering analysis in D, E and F.

B) Heatmap of the top 5 markers for each cluster.

C) Photoreceptor transcript expression projected onto the TSNE, as used for selecting clusters for further analysis.

D) TSNE plot of E18 photoreceptors after sub clustering analysis. Clusters are assign according to markers shown in E and F.

E) Heatmap op the top 5 markers for each photoreceptor cluster.

F) Transcript expression projected onto the TSNE plot, as used for photoreceptor cluster assignment.
